# Supplementary material for: Attractive targeted sugar baits for malaria control in western Kenya (ATSB-Kenya) – Effect of ATSBs on epidemiologic and entomologic indicators: A Phase III, open-label, cluster-randomised, controlled trial
Source: PLOS Glob Public Health. 2025 Jun 26;5(6):e0004230. doi: 10.1371/journal.pgph.0004230 (PMC12200848; doi:10.1371/journal.pgph.0004230)
Supplement: S1 Appendix — Detailed description of procedures used for malaria parasite detection using thick and thin blood smears, including staining procedures, parasite counting methodology, and quality control measures with independent readings by certified microscopists. (DOCX) [file pgph.0004230.s001.docx]

**Supplemental files**

**S1 Appendix – Malaria microscopy procedures**

For microscopy, a thick and thin blood smear were prepared on the same slide by study clinicians. Slides were transported to the Kenya Medical Research Institute (KEMRI) / Centre for Global Health Research laboratory in Siaya county for microscopy. Slides required for patient care were stained with 10% Giemsa for 15 minutes, while non-urgent smears were stained with 3% Giemsa for 1 hour. The thick blood smear was used to detect parasites and determine the parasite count, if <100 parasites per high power field (hpf) were seen. If no asexual or sexual forms of the parasites were seen after 2500 WBCs or 200 hpfs, the slide was declared negative. If parasites were seen before 500 WBCs or 40 fields were counted, counting continued up to 500 WBCs or 40 fields. If parasites were seen after 500 WBCs or 40 fields had been counted, reading was stopped after all the parasites and/or WBCs on the field were counted. For slides with >100 parasites/hpf, the thin blood smear was used for species identification and to determine parasite count. Parasitized RBCs along with RBCs were counted until a total of 2000 RBCs were counted. RBCs with more than one parasite or with multinucleate forms as 1 (one) infected RBC were counted, but parasites of different species were not counted separately. All slides were read twice by two independent malaria microscopy certified readers, and a third independent reviewer.
