# Supplementary material for: Attractive targeted sugar baits for malaria control in western Kenya (ATSB-Kenya) – Effect of ATSBs on epidemiologic and entomologic indicators: A Phase III, open-label, cluster-randomised, controlled trial
Source: PLOS Glob Public Health. 2025 Jun 26;5(6):e0004230. doi: 10.1371/journal.pgph.0004230 (PMC12200848; doi:10.1371/journal.pgph.0004230)
Supplement: S1 Table — Summary of the cohort recruitment process comparing intervention and control arms, including children screened, reasons for declining participation, exclusion criteria, and final enrolment numbers, with age stratification. (DOCX) [file pgph.0004230.s002.docx]

**Supplemental files**

## S1 Table – Cohort study recruitment

|  | **Overall** | | **Intervention (ATSB)** | | **Control** | |
| --- | --- | --- | --- | --- | --- | --- |
|  | **n** | **%** | **n** | **%** | **N** | **%** |
| Children on recruitment list (N) | 4,509 |  | 2,257 |  | 2,252 |  |
| Children not contacted | 804 | 17.8 | 399 | 17.7 | 405 | 18.0 |
| Children screened | 3,705 | 82.2 | 1,858 | 82.3 | 1,847 | 82.0 |
| Declined to participate | 220 | 5.9 | 111 | 6.0 | 109 | 5.9 |
| Reasons declined |  |  |  |  |  |  |
| ―Not interested in the study | 102 | 46.4 | 47 | 42.3 | 55 | 50.5 |
| ―No reason stated | 103 | 46.8 | 57 | 51.4 | 46 | 42.2 |
| ―Not comfortable with sample collection (blood draws) | 6 | 2.7 | 3 | 2.7 | 3 | 2.8 |
| ―Other* | 9 | 4.1 | 4 | 3.6 | 5 | 4.6 |
| Children excluded | 523 | 14.1 | 250 | 13.5 | 273 | 14.8 |
| Reasons for exclusion |  |  |  |  |  |  |
| ―Not a resident of a household within the core area | 392 | 75.0 | 185 | 74.0 | 207 | 75.8 |
| ―Not of appropriate age (≥ 1y & < 15y at enrolment) | 64 | 12.2 | 30 | 12.0 | 34 | 12.5 |
| *―Enrolled in prior ATSB cohort* | 21 | 4.0 | 10 | 4.0 | 11 | 4.0 |
| ―Confirmed or suspected pregnancy | 0 | — | 0 | — | 0 | — |
| ―Taking daily cotrimoxazole prophylaxis | 33 | 6.3 | 19 | 7.6 | 14 | 5.1 |
| ―Known sickle cell disease | 8 | 1.5 | 3 | 1.2 | 5 | 1.8 |
| ―Contraindication to AL | 0 | — | 0 | — | 0 | — |
| ―Currently enrolled in another interventional study | 5 | 1.0 | 3 | 1.2 | 2 | 0.7 |
| Children enrolled | 2,962 | 80.0 | 1,497 | 80.6 | 1,465 | 79.3 |
| ―Aged 1 to <5 years | 784 | 26.5 | 384 | 25.7 | 400 | 27.3 |
| ―Aged 5 to <15 years | 2,178 | 73.5 | 1,113 | 74.4 | 1,065 | 72.7 |

** Other reasons: child has previous burn injury (n=1), twins, declined unless both could participate (n=1), parent unable to consent (n=1), child has liver disease, parents uncomfortable (n=1), child bereaved due to loss of parent (n=1), child only available on weekends (n=1), child in grade 8 (n=2)*
