# Supplementary material for: Attractive targeted sugar baits for malaria control in western Kenya (ATSB-Kenya) – Effect of ATSBs on epidemiologic and entomologic indicators: A Phase III, open-label, cluster-randomised, controlled trial
Source: PLOS Glob Public Health. 2025 Jun 26;5(6):e0004230. doi: 10.1371/journal.pgph.0004230 (PMC12200848; doi:10.1371/journal.pgph.0004230)
Supplement: S2 Table — Comprehensive listing of serious adverse events occurring during the study, including patient demographics, clinical descriptions, severity assessment, relationship to intervention, and outcomes. (DOCX) [file pgph.0004230.s003.docx]

**Supplemental files**

## S2 Table – Serious adverse events & deaths

|  | **Cohort number** | **Study arm** | **Patient age (years)** | **Event description** | **Maximum**  **severity** | **Relationship to ATSB** | **Outcome** |
| --- | --- | --- | --- | --- | --- | --- | --- |
| 1 | 1 | Intervention | 2 | Severe malaria & tonsillitis | Severe | Not related | Resolved |
| 2 | 2 | Intervention | 7 | Severe malaria & pneumonia | Severe | Not related | Resolved |
| 3 | 1 | Control | 4 | Road traffic accident & head injury | Life-threatening | Not related | Fatal |
| 4 | 1 | Control | 5 | Severe malaria | Severe | Not related | Resolved |
| 5 | 2 | Control | 2 | Severe malaria, anaemia & acute kidney failure | Life-threatening | Not related | Fatal |
| 6 | 1 | Intervention | 4 | Severe malaria | Severe | Not related | Resolved |
| 7 | 2 | Intervention | 1 | Febrile convulsions | Severe | Not related | Resolved |
| 8 | 1 | Intervention | 14 | Severe malaria | Severe | Not related | Resolved |
| 9 | 1 | Control | 3 | Severe malaria | Severe | Not related | Resolved |
| 10 | 1 | Control | 2 | Severe malaria & anaemia | Severe | Not related | Resolved |
| 11 | 2 | Intervention | 10 | Severe malaria | Severe | Not related | Resolved |
| 12 | 2 | Control | 3 | Severe malaria | Life-threatening | Not related | Fatal |
| 13 | 1 | Intervention | 14 | Soft tissue infection (left arm) | Severe | Not related | Resolved |
| 14 | 2 | Intervention | 5 | Gastroenteritis | Severe | Not related | Resolved |
| 15 | 3 | Intervention | 2 | Severe malaria with convulsions | Severe | Not related | Resolved |
| 16 | 3 | Intervention | 12 | Severe malaria | Severe | Not related | Resolved |
| 17 | 3 | Control | 2 | Severe malaria, brain atrophy with possible  Guillain Barre syndrome, perianal ulcers | Severe | Not related | Fatal |
| 18 | 3 | Control | 2 | Brain atrophy with possible Guillain barre  syndrome, perianal ulcers, malaise, death | Life-threatening | Not related |  |
