# Supplementary material for: Attractive targeted sugar baits for malaria control in western Kenya (ATSB-Kenya) – Effect of ATSBs on epidemiologic and entomologic indicators: A Phase III, open-label, cluster-randomised, controlled trial
Source: PLOS Glob Public Health. 2025 Jun 26;5(6):e0004230. doi: 10.1371/journal.pgph.0004230 (PMC12200848; doi:10.1371/journal.pgph.0004230)
Supplement: S3 Table — Comparison of female Anopheles mosquito parity rates between intervention and control arms, including collections over 24 months of follow-up, with statistical analyses presented as both unadjusted and adjusted odds ratios. The adjusted analysis included fixed effects for collection location (indoors vs. outdoors), time since intervention and calendar month as a seasonality adjustment. (DOCX) [file pgph.0004230.s004.docx]

**Supplemental files**

**S3 Table – Additional parity outcomes**

| **Indicator** | **Study arm** | **Mean total number**  **dissected by cluster† (Min – Max)** | **Mean number dissected by month (Min – Max)** |  | **Unadjusted** | | **Adjusted*** | |
| --- | --- | --- | --- | --- | --- | --- | --- | --- |
|  |  |  |  | **n/N (%)** | **OR (95% CI)** | **p-value** | **OR (95% CI)** | **p-value** |
| Parity (Female *Anopheles spp.*) | Control | 299·88 (8–1183) | 99·96 (8–266) | 1973/2399 (82·2) | Ref |  | Ref |  |
|  | Intervention (ATSB) | 507·25 (9–2731) | 169·08 (39–458) | 3579/4058 (88·2) | 1·34 (0·905 - 1·99) | 0·14 | 1·30 (0·87 - 1·94) | 0·201 |

*† Including collections over 24 months of follow-up*

** Adjusted analysis for parity included fixed effects for collection location (indoors vs. outdoors), time since intervention and calendar month as a seasonality adjustment.*
