# Supplementary material for: Attractive targeted sugar baits for malaria control in western Kenya (ATSB-Kenya) – Effect of ATSBs on epidemiologic and entomologic indicators: A Phase III, open-label, cluster-randomised, controlled trial
Source: PLOS Glob Public Health. 2025 Jun 26;5(6):e0004230. doi: 10.1371/journal.pgph.0004230 (PMC12200848; doi:10.1371/journal.pgph.0004230)
Supplement: S4 Table — Species-specific analysis of mosquito parity and density outcomes, comparing Anopheles funestus and Anopheles gambiae between control and intervention arms across different collection methods (HLCs, UVLTs, and CSS 70 UVLT), with associated statistical measures. (DOCX) [file pgph.0004230.s005.docx]

**Supplemental files**

**S4 Table – Parity and density outcomes (stratified by species)**

| **Indicator** | **Study arm** |  | **Unadjusted** | | **Adjusted** | |
| --- | --- | --- | --- | --- | --- | --- |
|  |  | **n/N (%)** | **OR (95% CI)** | **p-value** | **OR (95% CI)** | **p-value** |
| Parity *(An funestus)** | Control | 1388/1657 (83·8) | Ref |  | Ref |  |
|  | Intervention (ATSB) | 3228/3632 (88·9) | 1·32 (0·82–2·12) | 0·25 | 1·31 (0·833–2·06) | 0·24 |
| Parity *(An gambiae)** | Control | 585/742 (78·8) | Ref |  |  |  |
|  | Intervention (ATSB) | 351/426 (82·4) | 1·25 (0·83–1·9) | 0·29 | 1·20 (0·744–1·93) | 0·46 |
|  |  | **Cluster Median (IQR) †** | **IRR (95% CI)** | **p-value** | **IRR (95% CI) ‡** | **p-value** |
| Density by HLCs *(An funestus)* | Control | 1·11 (0·78–4·19) | Ref |  | Ref |  |
|  | Intervention (ATSB) | 0·607 (0·28–9·17) | 0·67 (0·05–8·22) | 0·75 | 0·69 (0·06–8·54) | 0·77 |
| Density by HLCs *(An gambiae)* | Control | 0·89 (0·4–1·42) | Ref |  | Ref |  |
|  | Intervention (ATSB) | 0·366 (0·27–1·65) | 0·76 (0·22–2·63) | 0·66 | 0·79 (0·22–2·78) | 0·71 |
| Density by UVLTs *(An funestus)* | Control | 3·44 (2·18–5·76) | Ref |  | Ref |  |
|  | Intervention (ATSB) | 3·15 (1·23–17·6) | 0·88 (0·16–4·81) | 0·88 | 0·86 (0·158–4·72) | 0·87 |
| Density by UVLTs *(An gambiae)* | Control | 1·77 (0·98–2·62) | Ref |  | Ref |  |
|  | Intervention (ATSB) | 1·04 (0·6–3·48) | 0·82 (0·35–1·92) | 0·65 | 0·87 (0·35–2·21) | 0·78 |
| Density by CSS 70 UVLT (*An funestus)* | Control | 2·55 (0·6–7·92) | Ref |  | Ref |  |
|  | Intervention (ATSB) | 2·14 (0·4–14·8) | 1·06 (0·484–2·33) | 0·88 | 0·90 (0·58–1·4) | 0·637 |
| Density by CSS 70 UVLT (*An gambiae)* | Control | 1·1 (0·2–4·47) | Ref |  | Ref |  |
|  | Intervention (ATSB) | 0·8 (0·2–3·6) | 0†·87 (0·551–1·37) | 0·55 | 0·965 (0·635–1·46) | 0·866 |

** Parity analysis includes fixed effects for collection location (indoors vs. outdoors), time since intervention and calendar month as a seasonality adjustment*

*† Household density per night*

*‡ Adjusted analysis for density includes fixed effects for study arm (ATSB) + log(baseline anopheline species number) and random effects for cluster & round*
